# Quadrivalent Human Papillomavirus Vaccine Effectiveness after 12 Years in Madrid (Spain)

**DOI:** 10.3390/vaccines10030387

**Published:** 2022-03-03

**Authors:** Juan J. Hernandez-Aguado, Damián Ángel Sánchez Torres, Esther Martínez Lamela, Gema Aguión Gálvez, Eva Sanz Espinosa, Almudena Pérez Quintanilla, Daniela A. Martínez-Carrillo, Mar Ramírez Mena, Pluvio J. Coronado Martín, Ignacio Zapardiel, Jesús de la Fuente-Valero

**Affiliations:** 1 Gynecology Department, Infanta Leonor University Hospital, 28031 Madrid, Spain; damianangel.sanchez@salud.madrid.org (D.Á.S.T.); ester.martinez@salud.madrid.org (E.M.L.); gema.aguion@salud.madrid.org (G.A.G.); eva.sanz@salud.madrid.org (E.S.E.); aperezquin.hcsc@salud.madrid.org (A.P.Q.); danielaabsara.martinez@salud.madrid.org (D.A.M.-C.); delavalero@gmail.com (J.d.l.F.-V.); 2 Gynecology Oncology Unit, Institute of Women’s Health, San Carlos Clinical Hospital (IdISSC), Complutense University, 28040 Madrid, Spain; mariamar.ramirez@salud.madrid.org (M.R.M.); pluviojesus.coronado@salud.madrid.org (P.J.C.M.); 3 Gynecologic Oncology Unit, La Paz University Hospital-IdiPAZ, 28046 Madrid, Spain; ignaciozapardiel@alumni.harvard.edu

**Keywords:** HPV, vaccine, quadrivalent vaccine

## Abstract

A fully government-funded human papillomavirus (HPV) vaccination program started in 2007 in Spain (only 11–14-year-old girls). The first of those vaccinated cohorts, with the quadrivalent vaccine (Gardasil), turned 25 years old in 2018, the age at which cervical cancer screening begins in Spain. The current study could provide the first evidence about the effectiveness of the quadrivalent vaccine against HPV in Spain and the influence of age of vaccination. The present ambispective cohort study, which was conducted on 790 women aged 25 and 26 years old, compares the rate of HPV prevalence and cytologic anomaly according to the vaccination status. The overall infection rate was 40.09% (vaccinated group) vs. 40.6% (non-vaccinated group). There was a significant reduction in the prevalence of HPV 6 (0% vs. 1.3%) and 16 (2.4% vs. 6.1%), and in the prevalence of cytological abnormalities linked to HPV16: Atypical Squamous Cells of Undetermined Significance (ASCUS) (2.04% vs. 14%), Low-grade Squamous Intraepithelial Lesions (LSIL) (2.94% vs. 18.7%) and High-grade Squamous Intraepithelial Lesion (HSIL) (0% vs. 40%), in the vaccinated group vs. the non-vaccinated group. Only one case of HPV11 and two cases of HPV18 were detected. The vaccine effectively reduces the prevalence of vaccine genotypes and cytological anomalies linked to these genotypes.

## 1. Introduction

The expected benefits of vaccination against human papillomavirus (HPV) infection are a reduction in the prevalence of infection, cytological abnormalities in the cervix and genital warts. The effectiveness of a population-based vaccination program with the quadrivalent HPV vaccine has been investigated previously [1,2,3,4,5,6].

In Spain, a government-funded program with the quadrivalent vaccine (Gardasil^®^, covering HPV types 6-11-16-18) was established in 2007 for 11–14-year-old girls. Vaccine genotypes 16 and 18 cause preneoplastic lesions and cervical cancer, and vaccine genotypes 6 and 11 cause genital warts [1,4,5].

In 2018, the first cohort of vaccinated women was screened at the age of 25 years old, the age when the national screening program against cervical cancer begins in Spain [7].

Spanish data on the effectiveness of the quadrivalent vaccine is very scarce. One study published in 2017 reported an effectiveness of 77% after three doses of the vaccine against genital warts [8]. Another study published in 2020 suggests that a high vaccine uptake with quadrivalent vaccine not only provides direct benefits against genital warts in the vaccinated cohorts, but also extends protection through a herd effect to unvaccinated men [9]. In Spain, no data is available regarding the effectiveness after a 12-year follow-up on cytological abnormalities.

Our aim was to compare the prevalence of HPV infection and cervical cytological abnormalities among 25–26-year-old women previously vaccinated with the quadrivalent HPV with non-vaccinated women. In addition, we will analyze the effect of age on the HPV infection and cytological abnormality rates. 

## 2. Materials and Methods

### 2.1. Study Design

This study is an ambispective cohort study, stratified into two groups (women vaccinated against HPV and unvaccinated women).

Population: women living in Madrid (Spain), aged 25 to 26 years between October 2018 and October 2019 who were vaccinated with Gardasil in the systematic vaccination program (girls 11 to 14 years old) started in 2007, or in later years. At the beginning of the study, the participants had already had sex and had a cervix, females that had undergone a hysterectomy were excluded from the study.

The final number of studied women was 790 (376 women unvaccinated, and 414 women vaccinated with 3 doses).

The sample size calculation took into account the prevalence of HPV and cytological abnormalities, the proportion of women vaccinated and unvaccinated against HPV and an alpha and beta risk of 0.05/0.2, respectively. The standard deviation is assumed to be 5. A drop-out rate of 0% has been anticipated, as the study has no follow up.

The women who were candidates to be included in the study were identified from the data provided by the corresponding Public Health Department (vaccination and census data). We received data for identification, date of birth and the date on which the different doses of the vaccine were administered in those who were vaccinated.

Recruitment: Administrative staff contacted the women by phone, in a consecutive manner, from the hospital data bases and the Regional Vaccination Registry until reaching the required sample size.

### 2.2. Liquid-Based Cytology and HPV Testing

On the first visit, a midwife performed an epidemiological questionnaire and collected a cervical sample with the device Cervibrush (cervical device) (AMD ESAFARMA, Terontola, Italy) and stored it in Thinprep solution (Hologic Iberia, Madrid, Spain) for liquid-based cytology and HPV testing. 

Results were managed according to the prevention of cervical cancer AEPCC guideline in Spain [10].

The detection and genotyping of HPV was done in vitro by CLART HPV4 (PCR multiplex and low density arrays ((Genomina S.A.U., Madrid, Spain) which detects 35 HPV genotypes: HPV high risk (HR-HPV) (16, 18, 31, 33, 35, 39, 45, 51, 52, 56, 58, 59, 66, 68), probable high risk (26, 53, 73, 82) and HPV low risk (LR-HPV) (6, 11, 40, 42, 43, 44, 54, 61, 62, 70, 71, 72, 81, 83, 84, 85, 89). The test has a diagnostic sensitivity and specificity of 98.2% and 100%, respectively, and an analytical specificity of 100%. The analytical sensitivity of this test is 100% when the number of copies is 1.000 or 10.000 depending on the type of HPV.

Cytological studies were done by a cytotechnician and pathologists using the Bethesda 2014 nomenclature without knowledge of the HPV test.

### 2.3. Data Analysis

Quantitative variables are expressed with their mean and standard deviation (SD).

The association of qualitative variables between the two study groups was compared using the chi-square or Fisher exact test. Quantitative variables were compared by the Student’s *t*-test or the Mann–Whitney U test according to the distribution. For all tests, a *p* value less than 0.05 was considered as significant. The statistical study was done with the program IBM-SPSS version 21.

All of the participants signed the informed consent document. The study was approved by the Ethics Committee of Hospital Gregorio Marañón (protocol code 95/19; 6 May 2019).

## 3. Results

### 3.1. Population Characteristics

A total of 48% (378/792) of the participants were not vaccinated (NVG), and 52% (414/792) were vaccinated against HPV (VG). 

The mean age of the women included in the study was 25.10 ± 0.73 (24.6 ± 0.66 in VG vs. 25.5 ± 0.50 in NVG *p* = 0.0001) (asymmetric distribution).

In total, 80.1% (332/414) of vaccinated women received the first dose when they were 14 years old or younger, and the remaining 19.8% (82/414) received it when they were 15 years old or older. In VG at 14 or younger, only 0.91% (3/328) were sexually active at the time of vaccination. On the other hand, in VG at 15 or older, 38.46% (30/78) were sexually active at the time of vaccination (four individuals were missing to report on this aspect). Other descriptive variables analyzed are shown in Table 1.

### 3.2. HPV Infection Prevalence

Regardless of their vaccination status, 40.4% (319/790) of the participating women were positive for HPV, and the global prevalence of HR-HPV infection was 29.2% (231/790).

There are no statistically significant differences in the overall HPV infection rate between VG and NVG (40.09% (166/414) vs. 40.6% (153/376); *p* = 0.88), neither are there any when analyzing the percentage of HR-HPV infections: 28.6% (118/296) in VG vs. 30.5% (113/263) in NVG group, (*p* = 0.63) (Table 2). 

The overall rate of coinfections was 18.3% (145/790), regardless of vaccination status. There were no statistically significant differences in the rate of coinfections between VG and NVG against HPV 17.8% (74/414) vs. 18.8% (71/376); *p* = 0.94 (Table 3).

Women vaccinated at 14 years old or younger had a prevalence of HR-HPV of 24.4% (81/332), while in women vaccinated at 15 years old or older the prevalence was 45.1% (37/82) [OR 0.39 (95%CI: 0.23–0.64) *p* = 0.0003]. 

Regardless of the vaccination status, the five most prevalent HR-HPV types were 51 (5.8%, 46/790), 39 (4.65%, 36/790), 66 (4,2%, 33/790), 16 (4.2%, 33/790) and 52 (4.2%, 33/790). Among the HPV positive population, HPV16 was 10.82% (33/314) and rose to 14.6% (33/231) among the HR-HPV population. The global prevalence of HPV-LR types 6, 11 and HR-HPV type 18 was 0.6 (5/790), 0.1 (1/790) and 0.3% (2/790), respectively. 

When analyzing the genotypes targeted by the quadrivalent vaccine, a statistically significant reduction (*p* = 0.009) was shown for HPV 16 between VG vs. NVG (6.1% vs. 2.4%, respectively) (OR 2.63 (95%CI: 1.23–5.60)), which represents a reduction of 60%. Additionally, a significant reduction (*p* = 0.01) in the prevalence of HPV-LR 6 (1.3% vs. 0%) was observed, which represents a reduction of 100%, although the number of positives was very low. No significant differences were found for HPV 18, 31, 33 or 45. All results can be seen in Table 4.

### 3.3. Cytological Results

A total of 78.9% (617/782) of the total women studied had a normal cytological study, compared to 21% (165/782) with abnormalities: Atypical Squamous Cells of Undetermined Significance (ASCUS. 55.15% (91/165)); Low-Grade Squamous Intraepithelial Lesion (LSIL. 40% (66/165)) and High-Grade Squamous Intraepithelial Lesion (HSIL. 4.84% (8/165)). In VG, the rate of global cytological alterations was 21% (86/409), while in NVG it was 21.1% (79/373) OR: 1 (95%CI: 0.71–1.42). There were also no statistically significant differences when comparing the rates of ASCUS, LSIL and HSIL between the vaccinated and the non-vaccinated group (Table 5).

Among HR-HPV-positive women, the rate of cytologic abnormalities was 42.1% (96/228), compared to 12.4% (69/554) among HR-HPV-negative women OR: 5.11 (95% CI: 3.55–7.35; *p* = 0.0001) (Table 6).

The most frequent genotype detected in abnormal cytology in NVG was HPV 16 (17.72%. 14/79) and HPV 51 in VG (17.44%. 15/86). However, the presence of HPV 16 in abnormal cytology in VG was only 2.32% (2/86) (*p* = 0.001). For the remaining HR-HPV, there was no significant differences, except for genotype 56 for normal cytology, in which the frequency was significantly lower in the vaccinated group compared to the non-vaccinated (0.61% vs. 3.06%. *p* = 0.02) (Table 7). 

The ≤14 years old vaccinated subgroup had a prevalence of abnormal cytology of 19.5% (64/328), while the ≥15 years old vaccinated subgroup was 27.2% (21/77). OR 1.54 (95%CI: 0.87–2.73; *p* = 0.13). 

When we repeated the sub-analysis on vaccination age and rate of ASCUS and LSIL, we did not find significant differences for ASCUS, showing an OR 0.7 (95%CI: 0.30–1.62), but for LSIL, the prevalence is the highest in the group vaccinated late (16.8% vs. 6.4%) with an OR 2.96 (95%CI: 1.41–6.23; *p* = 0.01), making it three times more likely to present an LSIL cytology if vaccination is performed after 15 years old.

The OR of having an altered cytology related to HPV 16 according to the vaccination status is 6.22 (95%CI: 1.06–36.21; *p* 0.04), which means that the NVG is six times more likely to have an altered cytology related to HPV 16 than the group that was vaccinated. 

Table 8 reflects vaccination status and cytological result by type of abnormality in relation to the presence of HPV16. Statistically significant differences are demonstrated in the specific analysis for ASCUS (2% vs. 14.2%) (1/49 vs. 6/42) with *p* 0.029, and LSIL (2.9% vs. 18.7%) (1/34 vs. 6/32) with *p* = 0.037. There was no HSIL in VG compared to two cases of HSIL in NVG (*p* = 0.206). 

## 4. Discussion

In this study we show that vaccination against HPV at an early age (11–14 years old) in a cohort of Spanish girls is effective, diminishing the prevalence of HPV 6 and 16, as well as of cytological abnormalities.

The overall prevalence of HPV infection in our study is 10% higher than that described in other reports previously published in Spain [11]. In contrast to what has been published by Purriños et al., in a study carried out in a Spanish female population [12], we found no difference in overall HPV prevalence between VG and NVG.

Additionally, in a similar report in the culturally close Italian population, Carozzi et al. (2016) found a HR-HPV infection prevalence 8% lower than we do for a comparable age group [13]. 

In our study, NVG presents a lower use of condoms vs. VG (significant differences), which seems to indicate that women belonging to VG present greater awareness and take more protective measures against HPV. In both groups, a high number of sexual partners is observed, suggesting a change in sexual habits leading to greater promiscuity, which could explain the high global prevalence of HPV infection found. Although there is a tendency for vaccinated and unvaccinated women to have different habits, there are parameters of sexual habits that have not been collected in the study and therefore cannot be evaluated, constituting a limitation of the study.

Although no statistically significant differences were found between the vaccinated and non-vaccinated population regarding the overall rate of HPV infection in our study, Steben’s review carried out in Canada showed an even higher prevalence of HPV infection in the unvaccinated population (47.2%), meanwhile the prevalence rate for the vaccinated individuals is similar to what we report (36.1%) [14].

Our study reveals that there were differences in the detection rate of HR-HPV between the group of normal and altered cytology regardless of the vaccination status, which is similar to that described by Castellsagué [11] and the HPV Center for Spain [15] for a similar age group. 

Critically, however, we show that HR genotype 16, which is highly prevalent in the unvaccinated population (6.1%) is found in just 2.4% of the vaccinated individuals, being displaced from the top five most prevalent genotypes found in this experimental group, undoubtedly due to the protective effect of the vaccine. Accordingly, Garland SM [16] indicates approximately a 90% reduction in the prevalence of genotypes 6, 11, 16 and 18 with the vaccine Gardasil. In detail, we assess a prevalence reduction of up to 66% for genotype 16. Although remarkable and similar to that reported by Steben in Canada [14], this reduction is somewhat less than indicated by Tabrizi [3] in 2014 in similar ages (80%). 

On the other hand, we found a prevalence decrease of nearly 100% for genotype HPV 6, which is even higher than that also reported by Tabrizi [3] (84%).

Remarkably, we report no significant differences in the global HPV infection rate nor in global infection by high-risk HPV between the vaccinated and the non-vaccinated groups, coinciding with that reported by Carozzi et al. [13]. This can be explained by the high prevalence of other types (both high and low risk) that counteract the decrease demonstrated for genotypes HPV 6 and 16. When considering the joint prevalence of all four vaccine genotypes (HPV 6, 11, 16 and 18), we see a 70% decrease for the vaccinated group compared to the unvaccinated counterpart, which is somewhat lower than what Tabrizi and Markowitz reported in Australian and U.S. populations, respectively [3,17], but in line with the prevalence reductions shown by Chow (68%) [18] and Steben (73%) [14] in Australian and Canadian populations, respectively. When considering genotypes HPV16 and 31, 33 and 45 we also find an incidence reduction close to what was reported by the latter author. Purriños et al. reported for this group of genotypes a significant decrease in the vaccinated group (8.4% vs. 1.1%), although the vaccine used in their study was Cervarix [12]. 

A specific subanalysis focused on HPV-16 regarding the age at which the vaccine was received suggest that people vaccinated at age 15 or older could be more likely to be infected with HPV16 than those vaccinated at age 14 or younger. This could explain why 39.47% (33/76) of women who were vaccinated at 15 years of age or older had initiated sex prior to administration. These data, which suggest the importance of getting vaccinated at an early age, ideally before being exposed to HPV, are consistent with the report by Lei et al. [1], which indicates that the greatest reduction in risk is associated with a younger age at the start of vaccination. This is also in line with previous findings which showed a lower risk of genital warts and high-grade cervical lesions with HPV vaccination [19,20,21,22], supporting the recommendation of administering the quadrivalent HPV vaccine prior to exposure to HPV infection in order to achieve the most substantial benefit, since vaccination has no therapeutic effect against pre-existing HPV infection [23,24]. These findings are also confirmed by studies published in Sweden and Denmark in relation to protection against cervical cancer [1,2]. This should not, however, prevent the recommendation of vaccination against HPV for adult women, due to the benefits that the vaccination can bring them [25].

In our study, we found a global rate of cytological alterations higher than that reported by the College of American Pathologists (CAP) (ASCUS 4.3; LSIL 2.5, HSIL 0.5) [26], and by the Spanish Association of Cervical Pathology and Colposcopy (AEPCC) (ASCUS 1.6; LSIL 1.3%; HSIL 0.7) [27], regardless of the vaccination status. Our higher prevalence could be explained because our study is limited to an age in which the prevalence of HPV infection is highest and, subsequently, also the related cytological abnormalities, while CAP and AEPCC publications include a much wider age range population. Similar to what was indicated by Carozzi [13], on the other hand, we found no significant differences in the overall rate of cytological alterations between the vaccinated and non-vaccinated groups, regardless of HPV genotype. Additionally, no significant differences were found when the rate of ASCUS, LSIL and HSIL were separately analyzed for both groups. Perhaps our study is demographically limited, and perhaps with a higher n or at later ages, differences become evident for the population studied.

Importantly, when considering specifically HPV 16, we did find differences between the vaccinated and the non-vaccinated groups regarding the overall rate of cytological abnormalities present. Women vaccinated from 15 years of age are 1.5 times more likely to have an abnormal cytology with LSIL being almost three times more frequent. These findings are consistent with those reported by Castle et al. in 2019 [28], in which they find a greater reduction in high- and low-grade cytological lesions the lower the age at which the vaccine is administered, which translates to an increase in the absolute and relative risk of presenting these alterations as the vaccination age increases. This strongly underlines the importance of vaccination at an early age. Due to the low prevalence of HPV 16 in women with normal cytology (3%), this has little overall statistical effect. This finding is also consistent with what was published by the HPV Center for Spain in 2019 [15] (2.7%). In contrast, however, we found that the prevalence of HPV 16 in the case of abnormal cytology is below that indicated by the HPV Center of Spain [15] (12 vs. 40).

The presence of HPV 16, regardless of the vaccination status, is related to a greater risk of presenting an altered cytology compared to when HPV 16 is not detected. The presence of HPV 16 multiplies by 3.78 the probabilities of having an abnormal cytology when this group is compared to the rest of the studied population (including both negative HPV and other HPV genotypes), which stresses the importance of this genotype in the genesis of abnormal cytology.

In the unvaccinated population, there are six cytological abnormalities linked to HPV16 for each abnormality found in the vaccinated group which reinforces the efficacy of the vaccine in preventing cervical lesions of cervical cancer.

When we study the prevalence of ASCUS and LSIL related to HPV16, comparing the vaccinated and non-vaccinated groups, we found that both are significantly higher in the non-vaccinated. Differences in HSIL are also noted, but the low n of these cases means there is no statistical power to demonstrate differences. There was no HSIL in the vaccinated group versus two cases of HSIL in the non-vaccinated group. All of the above shows the clear effect of the protection developed by the vaccine. Not being vaccinated increases the probability of presenting an ASCUS or LSIL related to HPV 16 by five times. These data are consistent with what was reported by Ozawa [29].

The differences found in the prevalence of HR-HPV and HPV16, in the incidence of cytological ASCUS and LSIL, are significant when comparing the group with early vaccination (14 or less) with late vaccination (15 or more), always with more unfavorable data. In this last group, which supports the importance of being vaccinated before the start of sexual activity, it is considered that these differences are due to this factor since 39% of the patients vaccinated subsequently (15 years or older) were already sexually active before being vaccinated, so they had been exposed to the infection before being immunized against only 5% of women who were sexually active before being vaccinated in the early vaccination group (14 years or younger).

This study has some limitations related to a low value of n to evaluate some alterations (HSIL) that in themselves have a low prevalence. It would also have been necessary to expand this n to demonstrate differences with respect to less frequent vaccine genotypes (HPV 11 and 18). The non-differentiation in the vaccinated group of the age at which the vaccine was received may lead to a bias in the comparisons with the unvaccinated group given the lower effectiveness observed when the age of vaccination was 15 years or older.

Finally, the five high-risk HPV types included in the nonavalent vaccine, compared to the quadrivalent vaccine (HPV 31, 33, 45, 52, 58) are present in 14.43% (114/790) of our study population. Assuming 100% preventive efficacy of the nonavalent vaccine against these five types, the use of the vaccine could lead to a reduction of 14.43% in their prevalence and a reduction of 6.58% (52/790) in the rate of cytological alterations produced by them. 

## 5. Conclusions

The vaccine effectiveness is demonstrated, showing a reduction in the prevalence of the HPV 6 and 16 vaccine genotypes in the group that was vaccinated as well as a reduction in cytological alterations related to HPV 16.

## Figures and Tables

**Table 1:** Descriptive data of the study participants. * Statistical significance. ^#^ Asymmetric distribution. ^1^ sexually transmitted infections.

Variables	Non Vaccinated Group (*n* = 376)	Vaccinated Group (*n* = 414)	*p*
Ethnicity			0.025 *
Caucasian	(277) 73.7%	(338) 81.5%	
Latin	(95) 25.2%	(75) 18.2%	
Black	(3) 0.8%	(0) 0%	
Asian	(1) 0.2%	(1) 0.2%	
Civil Status			0.011 *
Married	(28) 7.5%	(14) 3.39%	
Single	(348) 92.5%	(400) 96.6%	
Age at first sexual intercourse (years) ^#^	16.7 ± 2.1	17.1 ± 2	0.007 *
Sexual orientation			0.973
Heterosexual	(360) 95.7%	(396) 95.6%	
Homosexual	(5) 1.3%	(5) 1.2%	
Bisexual	(11) 2.9%	(13) 3.1%	
Lifetime number of sexual partners ^#^	5.3	5.1	0.886
0–5	(271) 72.07%	(294) 71.01%	
6–10	(69) 18.35%	(89) 21.49%	
11–20	(29) 7.71%	(28) 6.76%	
≥21	(7) 1.86%	(3) 0.72%	
Women with births ^#^	(58) 15.42%	(27) 6.52%	0.0001 *
Average number of births per woman ^#^	0.22 ± 0.56	0.08 ± 0.33	0.0001 *
Condom use			0.0001 *
Never	(148) 39.3%	(122) 29.4%	
Occasionally	(83) 21.9%	(57) 13.7%	
Half the time	(15) 4.1%	(21) 5.1%	
Most of the time	(42) 11.2%	(69) 16.5%	
Always	(88) 23.2%	(145) 35.1%	
Tobacco smoking status			0.375
Yes	(121) 32.1%	(120) 29%	
No	(255) 67.9%	(294) 71%	
History of other STIs ^1^			0.601
Yes	(20) 5.4%	(14) 3.5%	
Chlamydia (*n*)	7	4	
Gonococo (*n*)	1	1	
Genital Herpes (*n*)	9	8	
HIV (*n*)	1	1	
Trichomona (*n*)	2	0	
No	(356) 94.6%	(400) 96.5%	

**Table 2:** Prevalence of HPV and high-risk HPV infection according to vaccination status.

Vaccination Status	HPV+/HPV−	% Positives	HR-HPV+/HR-HPV−	% Positives
Vaccinated	166/248	40.09	118/296	28.6
Unvaccinated	153/223	40.6	113/263	30.5

**Table 3:** HPV co-infections and vaccination status.

	Not Vaccinated	Vaccinated	Total	
HPV test				
Negative	223	248		471
Positive	153	166		319
Monoinfection	82	92	174	
Coinfection	71	74	145	
Total	376	414		790

**Table 4:** Prevalence of HPV genotypes according to vaccination status.

HPV Genotype	Frequency *N* = 790	Overall (%) *N* = 790	Unvaccinated (*n*) *n* = 376	Vaccinated (*n*) *n* = 414	* p *
6	5	0.6	1.3% (5)	0% (0)	0.019
11	1	0.1	0.3% (1)	0% (0)	0.294
16	33	4.2	6.1% (23)	2.4% (10)	0.009
18	2	0.3	0.5% (2)	0% (0)	0.137
31	27	3.4	4% (15)	2.9% (12)	0.399
33	12	1.5	1.3% (5)	1.7% (7)	0.679
35	15	1.9	2.4% (9)	1.4% (6)	0.358
39	36	4.6	4.3% (16)	4.8% (20)	0.698
45	12	1.5	2.1% (8)	1% (4)	0.183
51	46	5.8	4.8% (18)	6.8% (28)	0.236
52	33	4.2	3.5% (15)	4.3% (18)	0.801
56	19	2.4	3.4% (13)	1.4% (6)	0.066
58	30	3.8	3.5% (13)	4.1% (17)	0.634
59	32	4	3.2% (12)	4.8% (20)	0.243
66	33	4.2	3.7% (14)	4.6% (19)	0.543
68	12	1.5	1.3% (5)	1.7% (7)	0.679

The vaccinated subgroup ≤14 years old had a prevalence of HPV16 of 1.81% (6/331), while the vaccinated subgroup ≥15 years old was 5% (4/79). OR 2.88 (95%CI: 0.79–10.49; *p* > 0.10).

**Table 5:** Cytological results according to vaccination status.

Cytological Result	Global (%) (*n* = 782)	Unvaccinated (%) (*n* = 373)	Vaccinated (%) (*n* = 409)
Normal	617 (78.9)	294 (78.8)	323 (78.9)
Abnormal	165 (21)	79 (21.1)	86 (21)
ASCUS	91 (11.6)	42 (11.2)	49 (11.9)
LSIL	66 (8.4)	32 (8.5)	34 (8.3)
HSIL	8 (1)	5 (1.3)	3 (0.7)

**Table 6:** Cytological results by specific alteration and according to vaccination status.

Cytology Results			HR-HPV-Positive		Total
			Undetected	Detected	
Negative	HPV Vaccinated	No	229	65	294
		Yes	256	67	323
	Total		485	132	617
ASCUS	HPV Vaccinated	No	24	18	42
		Yes	31	18	49
	Total		55	36	91
LSIL	HPV Vaccinated	No	6	26	32
		Yes	7	27	34
	Total		13	53	66
HSIL	HPV Vaccinated	No	1	4	5
		Yes	0	3	3
	Total		1	7	8
Total	HPV Vaccinated	No	260	113	373
		Yes	294	115	409
	Total		554	228	782

**Table 7:** Cytological results according to present genotype (coinfections are not individualized) and according to vaccination status. * Statistical significance. ^&^ Non-calculable.

HPV Genotype	Normal Cytology (*n*)			Abnormal Cytology (*n*)		
	Vaccinated (323)	Not Vaccinated (294)	*p*	Vaccinated (86)	Not Vaccinated (79)	*p*
16	2.47% (8)	3.06% (9)	0.658	2.32% (2)	17.72% (14)	0.001 *
18	0% (0)	0.6% (2)	0.138	0% (0)	0% (0)	NC ^&^
31	1.54% (5)	3.74% (11)	0.087	8.13% (7)	5.06% (4)	0.429
33	0.61% (2)	1.36% (4)	0.349	5.81% (5)	1.26% (1)	0.119
35	1.54% (5)	2.04% (6)	0.517	1.16% (1)	3.79% (3)	0.272
39	3.09% (10)	2.72% (8)	0.782	11.62% (10)	10.12% (8)	0.757
45	0.30% (1)	1.70% (5)	0.079	3.48% (3)	3.79% (3)	0.916
51	3.71% (12)	3.40% (10)	0.834	17.44% (15)	10.12% (8)	0.175
52	3.09% (10)	3.40% (10)	0.831	8.13% (7)	6.32% (5)	0.655
56	0.61% (2)	3.06% (9)	0.022 *	4.65% (4)	5.06% (4)	0.902
58	1.85% (6)	2.04% (6)	0.869	11.62% (10)	8.86% (7)	0.559
59	4.02% (13)	2.38% (7)	0.250	6.97% (6)	6.32% (5)	0.868
66	3.09% (10)	2.04% (6)	0.410	10.46% (9)	10.12 (8)	0.943
68	1.54% (5)	1.02% (3)	0.563	2.32% (2)	2.53% (2)	0.931

**Table 8:** Vaccine status and cytological result by type of abnormality in relation to the presence of HPV16.

Vaccination Status			HPV16		Total
			No	Yes	
No	Cytology results	Normal	285	9	294
		ASCUS	36	6	42
		LSIL	26	6	32
		HSIL	3	2	5
	Total		350	23	373
Yes	Cytology results	Normal	315	8	323
		ASCUS	48	1	49
		LSIL	33	1	34
		HSIL	3	0	3
	Total		399	10	409
Total	Cytology results	Normal	600	17	617
		ASCUS	84	7	91
		LSIL	59	7	66
		HSIL	6	2	8
	Total		749	33	782

## Data Availability

Not applicable.
